# Blood hyperviscosity identification with reflective spectroscopy of tongue tip based on principal component analysis combining artificial neural network

**DOI:** 10.1186/s12938-018-0495-3

**Published:** 2018-05-10

**Authors:** Ming Liu, Jing Zhao, XiaoZuo Lu, Gang Li, Taixia Wu, LiFu Zhang

**Affiliations:** 1 Institute of Biomedical Engineering, Chinese Academy of Medical Sciences and Peking Union Medical College, Tianjin, 300192 China; 2 Institute of Chinese Medicine, Tianjin University of Traditional Chinese Medicine, Tianjin, 300193 China; 3 State Key Laboratory of Precision Measurement Technology and Instruments, Tianjin University, Tianjin, 300072 China; 4 Institute of Remote Sensing and Digital Earth, Chinese Academy of Sciences, Beijing, 100101 China

**Keywords:** Reflective spectroscopy, Noninvasive, Blood hyperviscosity diagnosis, Principal component analysis, Artificial neural network

## Abstract

**Background:**

With spectral methods, noninvasive determination of blood hyperviscosity in vivo is very potential and meaningful in clinical diagnosis. In this study, 67 male subjects (41 health, and 26 hyperviscosity according to blood sample analysis results) participate.

**Methods:**

Reflectance spectra of subjects’ tongue tips is measured, and a classification method bases on principal component analysis combined with artificial neural network model is built to identify hyperviscosity. Hold-out and Leave-one-out methods are used to avoid significant bias and lessen overfitting problem, which are widely accepted in the model validation.

**Results:**

To measure the performance of the classification, sensitivity, specificity, accuracy and F-measure are calculated, respectively. The accuracies with 100 times Hold-out method and 67 times Leave-one-out method are 88.05% and 97.01%, respectively.

**Conclusions:**

Experimental results indicate that the built classification model has certain practical value and proves the feasibility of using spectroscopy to identify hyperviscosity by noninvasive determination.

## Background

Blood hyperviscosity influences severely on human health. According to the reports, there is a growing awareness of blood hyperviscosity as the leading cause of cardiovascular disease [1–3]. Antonova et al. report increasing blood viscosity leads to vascular obstruction, and induces thrombosis and atherosclerosis [4]. And chronic blood hyperviscosity is a serious threat factor for human life. Therefore, the blood hyperviscosity diagnosis is a very important element to prevent and control the chronic vascular diseases. Currently, the diagnosis results of blood sample are provided by hemorheological instruments, and the relative procedure is still complex and time consuming. Furthermore, blood collection procedure may bring suffering to patients, and even lead to infection sometimes. So noninvasive and fast detection methods are required to be developed for hyperviscosity diagnosis. In the clinical environment, the integration of optical spectroscopy into disease examinations has the potential to substantially improve clinical practice [5, 6], and studies on spectroscopy used for clinical disease diagnosis has progressed rapidly. For example, Kan Lin et al. report a rapid fiber optic Raman spectroscopy for real-time in vivo detection of gastric intestinal metaplasia during clinical gastroscopy [7]. Albert et al. use mid-infrared and deported spectroscopy for septic arthritis diagnosis [8]. However, spectroscopy applied for blood hyperviscosity identification in vivo and noninvasively has not been reported. As we known, abnormal hemorheology is able to affect the state of human tongue, results in the changes of tongue features, such as tongue body color or presence of tongue coat, which provides significant information for human body health [9]. As a measurement site, Burmeister et al. propose a method of noninvasive blood glucose measurement by near infrared transmission spectroscopy across human tongues, and experiment results show that the tongue has more vascularity and less fatty tissue than the other sites such as the cheek, lower lips, upper lip, nasal septum, webbing tissue between the thumb and forefinger, which make the tongue become an excellent site for noninvasive disease diagnosis [10, 11]. Reasonably, the changes in the visible and near-infrared spectra at the tongue are able to reflect the blood viscosity information.

In this study, a new classification method is developed to identify blood hyperviscosity disease in vivo and noninvasively using human tongue reflective spectra bases on principal component analysis (PCA) combined with artificial neural network (ANN). To test the performance of the proposed method, the visible and near-infrared spectra experiment system is built, the spectra data is collected and blood sample is obtained from volunteer subjects. Experiment results are contrast to the blood analysis results, which demonstrate this method has the ability to extract blood viscosity information from reflective spectra. This paper is organized as follows: in “Collection and system”, the reflective spectra collection system and data collection are described briefly. In “Methods”, the procedure of the proposed method is presented. In “Experimental results”, the optic spectra data is analyzed by the proposed method and the performance of the method is investigated. At last, some discussions and conclusions are given in “Discussions” and “Conclusions”.

## Collection and system

### Data collection

A total of 67 male subjects (median age: 49; range 25–72 years) were willing to participate in this study, who had not taken any medication for at least 1 week and came in the morning after fasting period of 12 h. Reflectance spectra data was acquired at tongue tip from each subject, and then blood sample was obtained. Clinical hemorheology testing is carried out and the testing indices mainly include: blood viscosity (shear rates of 1, 5, 30, and 200 per s), erythrocyte sedimentation rate, hematocrit, relative index of blood viscosity at high shear rate, relative index of blood viscosity at low shear rate, erythrocyte sedimentation rate equation’s K, erythrocyte aggregation index, reduction viscosity at low shear rate, reduction viscosity at high shear rate, erythrocyte deformation index, erythrocyte rigidity index, and Casson viscosity. After that, clinical diagnosis results are provided by the experienced physician. Using clinical diagnosis results of the blood analysis as the standard, subjects were classified, health group (41 subjects) and blood hyperviscosity group (26 subjects). The experiment had obtained each subject’s consent, and in accordance with relevant laws.

### Measurement instruments

**Fig. 1:**
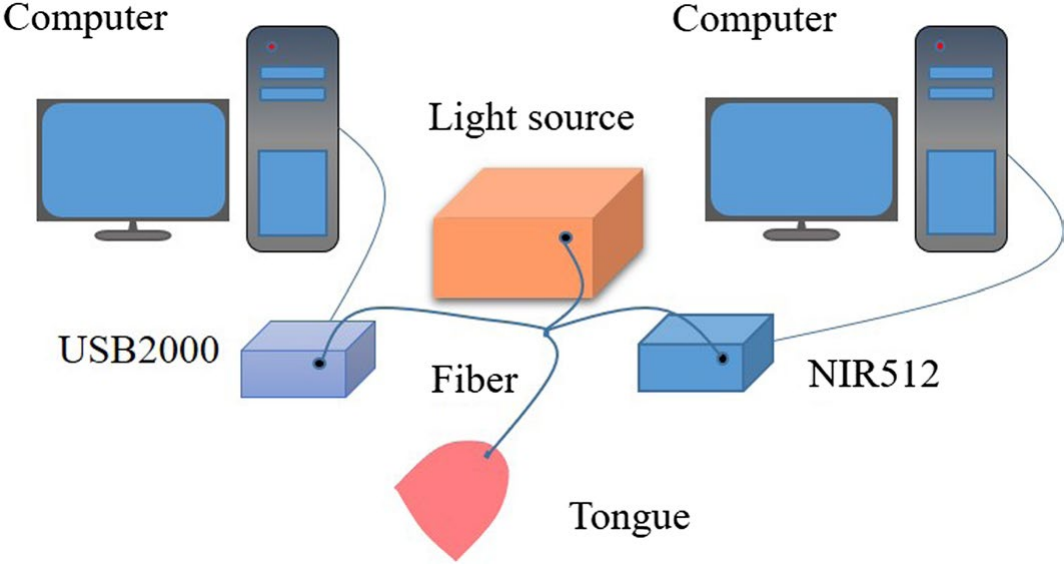
Experiment system schematic diagram. Two spectrometers connect to Tungsten-filament lamp through the optical fiber, and are controlled by two computers. Optical fiber probe is perpendicular to the tongue tip surface

Experiment system consists of computer, light source, spectrometer, and optical fiber. Two Dell computers (CPU: Intel core i5-4210 M, 2.60 GHz; RAM 4.00 GB, 64bit) are used as the processor for spectrometer control and data storage. 20 W Tungsten-filament lamp is used as the experiment light source, two spectrometers (USB2000 Ocean Optics, 462.87–1136.16 nm, and NIR512 Ocean Optics, 853.59–l737.26 nm) are used to collect tongue reflectance spectra. USB2000 is mainly used to obtain the visible spectroscopy, NIR512 is focused on acquiring near-infrared spectroscopy. The total wavelength region is 462.87–1737.26 nm, and the integration time is 3 and 35 ms, respectively. The total number of reflectance spectra data is 2558 bands obtained from each subject. Optical fiber connected light source and spectrometers is used to carry the incident and reflected light. Optical fiber probe is placed 10 mm above the tongue tip and perpendicular to the tongue tip surface. For each sample, the measurement number is 50 times at the same position. The experiment system is shown in Fig. 1.

**Fig. 2:**
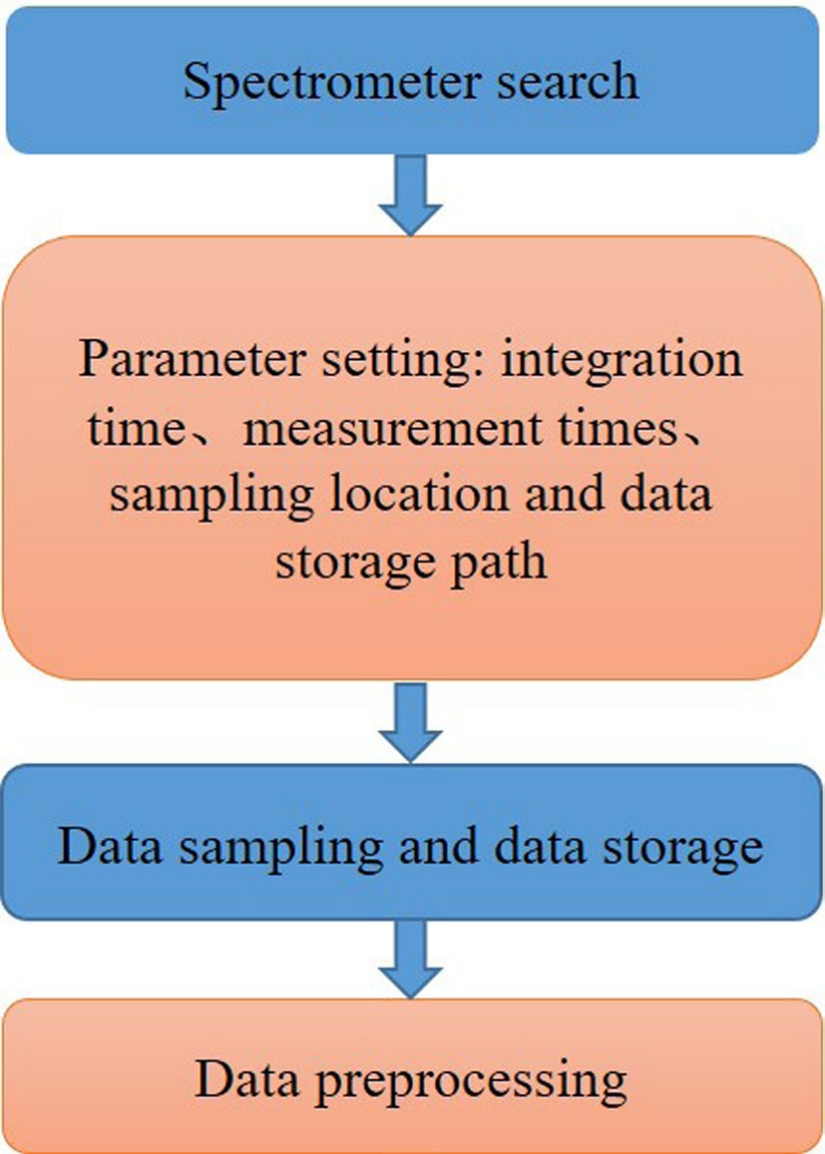
Flowchart of function module of the data acquisition system

We build homemade software for tongue reflectance spectra data acquisition and data storage. The homemade software is written by VB.net language. The spectrometer is connected to the computer via USB. The homemade software automatically recognizes the version of spectrometer, and then the operator can set integration time, measurement times, sampling location and data storage path on the front panel. The sample procedure and function module of the software are shown in Fig. 2.

## Methods

### Normalized reflectivity

The recorded spectra data is pre-processed to smooth the noise using the normalized reflectivity method, which is presented in formula (1).

1 $$R_{g} = R./\hbox{max} \left( R \right)$$

where $R_{g}$ is the normalized reflectivity, max(*R*) is the maximum of reflectivity in different wavelength. After normalized reflectivity, the spectral data will be sent to the artificial neural networks model as inputs.

### Principal component analysis

The collected spectra wavelength region is between 462.87 and 1737.26 nm. Wide wavelength region provide large amount of information, however, wide wavelength also result in increased difficulty and complexity of data analysis. In order to use less variable to take the place of the former variable and trim down the data dimension and redundancy, principal component analysis is used for data dimension reduction. PCA is considered as one of the most robust multivariate statistical methods of data analysis [12, 13]. The tongue normalized reflectivity $R_{g}$ is used as the input variable of PCA, and then the optimal number *P* of principal component *F*_*i*_ (*i *= 1,2,…,*P*) is determined which is based on the cumulative contribution rate of the principal components. The matrix dimension is trimmed down by PCA, and the PCA procedure is shown as follows:

1. Prepare training set, marked *n*. The number of spectral data is *sd*, marked *p *= *sd*, built n*p matrix and marked *X*;
2. Matrix *X* is standardized and marked *X*_*0*_;
3. Calculate correlation coefficient matrix *R*;
  $$R = \left( {r_{ij} } \right)_{sd \times sd}$$
  $$r_{ij} = \frac{{\mathop \sum \nolimits_{k = 1}^{n} \left( {x_{ki} - \bar{x}_{i} } \right)\left( {x_{kj} - \bar{x}_{j} } \right)}}{{\sqrt {\mathop \sum \nolimits_{k = 1}^{n} \left( {x_{ki} - \bar{x}_{i} } \right)^{2} \mathop \sum \nolimits_{k = 1}^{n} \left( {x_{kj} - \bar{x}_{j} } \right)^{2} } }}$$
4. Calculate characteristics root *λ*_*i*_ and the corresponding feature vector *a*_*i*_;
5. Extract principal components *F*_*i*_;
  $$F_{i} = a_{1i} X_{1} + a_{2i} X_{2} + \cdots + a_{Pi} X_{P } , i = 1, \cdots ,P$$
6. Calculate contribution rate and cumulative contribution rate of the principal components. Generally, when cumulative contribution rate reaches 85–95%, the corresponding principal component is extracted to represent the original information.

### Artificial neural network

After PCA, data dimension is trimmed down, on the premise of that the data information has been retained effectively. Principal component *F*_*i*_ (*i *= 1,2,…,*p*) is used as the input variables of classification model. Less input variables bring the complexity reduction of classification model. ANN is used to build the classification model in this work, which is established on the basis of modern neuroscience research. It uses large amount of processing unit to compose a complex model, and imitates human brain neural network structure and function. ANN has the function of self-organization, self-study, robustness, fault tolerance and nonlinear information processing and widely used in spectral analysis and identification [14, 15]. In this work, ANN is used to model the spectral data (inputs) and correlate it to clinical diagnosis result (outputs). ANN consists of a large number of units. The basic processing unit is a neuron, and it consists of input vector *X*, weight vector W, activation function $f\left( \cdot \right)$, bias parameter b and output vector *Y*. Mark inputs is *X*_*i*_ (*i *= 1,2,…,*p*). Output vector *Y* can be written as

$$Y = f\left( {W*X + b} \right) = f\left( {\sum W_{i} X_{i} + b} \right) i = 1,2, \ldots ,p$$

**Fig. 3:**
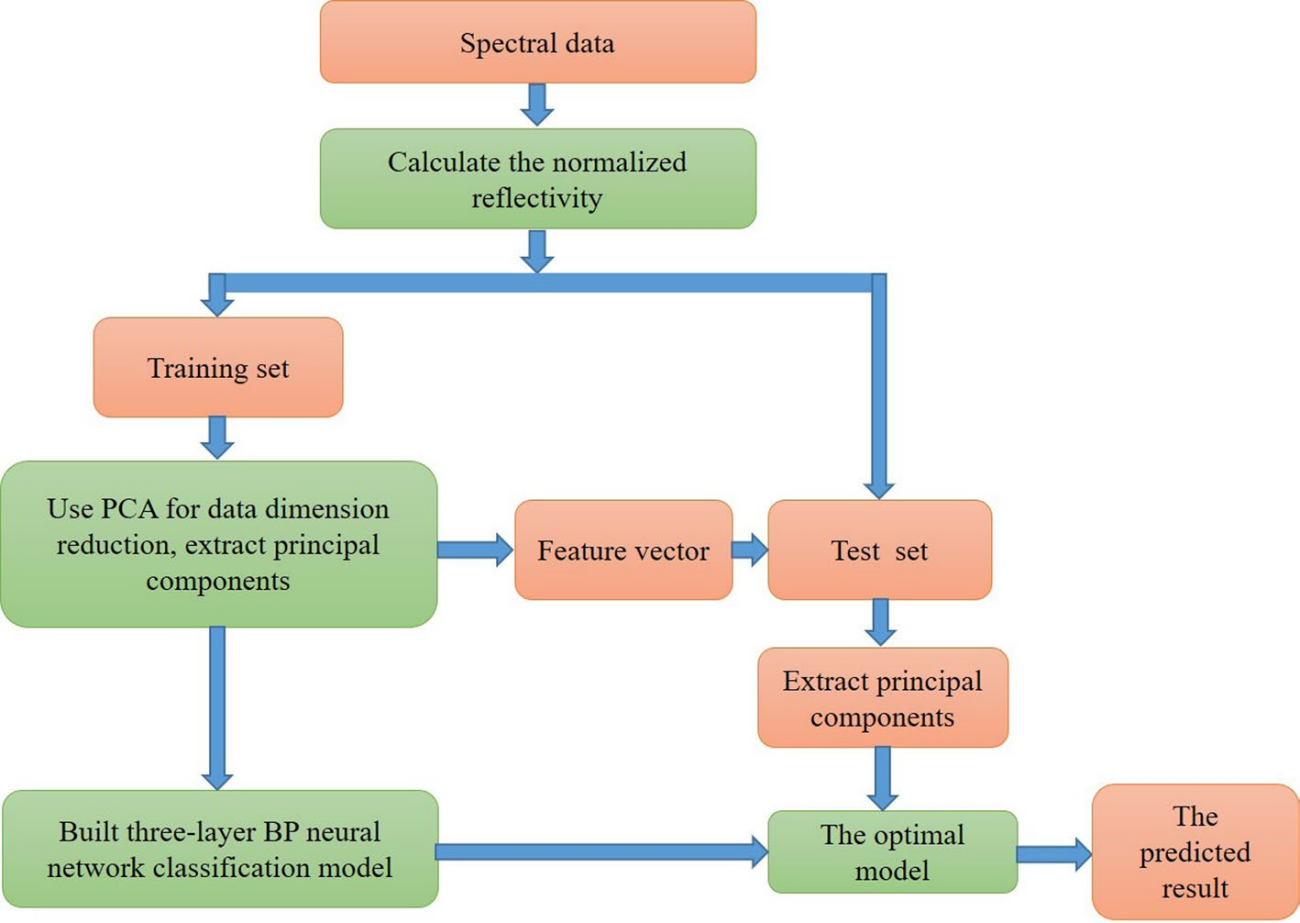
Block diagram of the tongue spectral data analysis

Set 1 represents health group and − 1 represents hyperviscosity group. When an unknown sample has entered into operation, if the output vector approximates to 1, the unknown sample is classified as healthy subject; if the output vector approximates to − 1, the unknown sample is classified as hyperviscosity subject. In this paper, a three-layer back-propagation (BP) neural network is used. According to empirical formula, the optimal number of neurons in hidden layer is determined by $\sqrt {n + m} + a$ [16]. Here, n is the number of the input layer neurons, namely, equal to the number of principal components determined by PCA; m is the number of the output layer neurons; a is an adjustable factor (range from 1 to 10, integer) and determined, while the mean square error between outputs and true values is less than the default. Tansig and tansig functions are used as the activation functions for hidden layer and output layer, respectively. Traingdm function is used as the training function. The weights and biases of the BP neural network are adjusted to make minimal the average MSE of BP neural network. The modeling process finishes, when the classification precision of this BP neural network diagnosis system is achieved (MSE = 0.001). Set the prediction deviation within ± 0.5 is right. Block diagram of the tongue spectral data analysis is as shown in Fig. 3.

### Cross validation

Hold-out and Leave-one-out cross validations are used to assess the performance of the proposed analysis method, which are widely accepted in the model validation. In the process of Hold-out method, the total data are divided into test set and training set in the ratio 3:7. Thereinto, 12 health and 8 hyperviscosity samples are used as test set at random, and the rest of data (nearly 70% of total data) are used as training set. To ensure the stability of the results, this analysis process is repeated 100 times, and the average of the results are mainly used to assess the performance of Hold-out method. In the process of Leave-one-out method, one sample is as test set and the rest of samples are as training set (66 samples), the whole process is repeated 67 times. And the average of the results is also used to assess the performance of Leave-one-out method.

## Experimental results

Since the whole blood is non-Newtonian liquid, and blood viscosity changes with shear rate. The normal range of low shear rates (1 1/s) and (5 1/s) are 17.63–21.35 and 8.31–9.95 mPa.S, respectively. The normal range of medium and high shear rates are 5.18–5.94 and 3.53–4.65. Blood viscosity under different shear rate is measured in the blood flow test of subjects. The blood viscosity distribution of hyperviscosity and health subjects is as shown in Tables 1 and 2.

**Table 1:** The whole blood viscosity distribution of hyperviscosity patients group under different shear rate

Shear rate	Blood viscosity distribution (mPa. S)	Mean value (mPa. S)	Standard deviation
(1 1/s)	21.15–26.6	23.22	1.699
(5 1/s)	10.09–12.83	10.87	0.8280
(30 1/s)	6.00–7.99	6.597	0.5546
(200 1/s)	4.58–6.29	5.11	0.4641

**Table 2:** The whole blood viscosity distribution of healthy group under different shear rate

Shear rate	Blood viscosity distribution (mPa. S)	Mean value	Standard deviation
(1 1/s)	16.34–21.42	19.47	1.716
(5 1/s)	7.59–9.82	9.097	0.7251
(30 1/s)	4.57–5.88	5.514	0.4240
(200 1/s)	3.53–4.66	4.268	0.3289

**Fig. 4:**
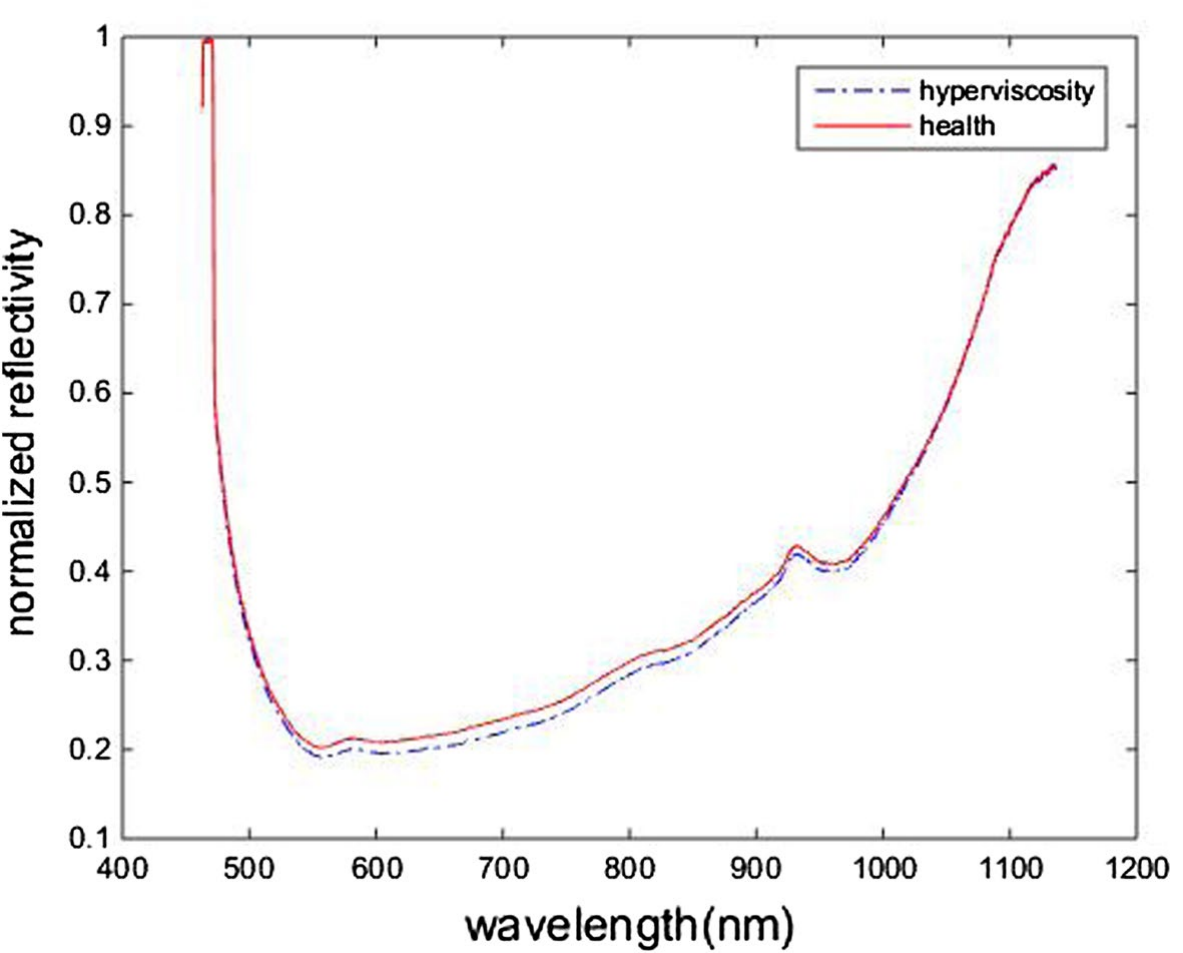
The average reflectance spectra of hyperviscosity and healthy subjects. The blue dotted line is the average reflectance spectra of hyperviscosity, and the red solid line is that of healthy subjects

**Fig. 5:**
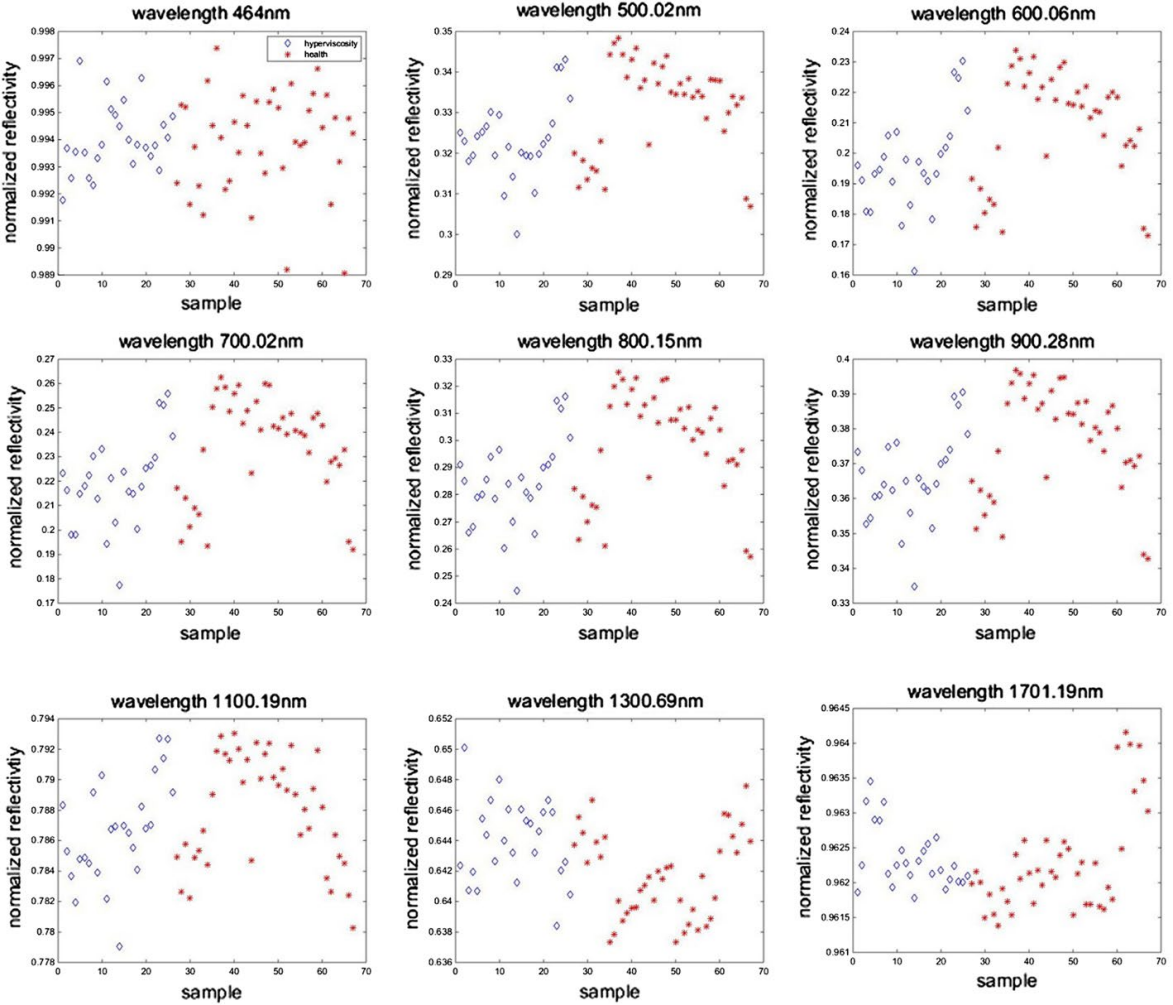
The normalize reflectivity scatterplots of each subject with the different wavelength, the blue diamond represents the hyperviscosity sample, and the red asterisk represents the health sample

As is known, the reflectance spectrum can present the property of subject tongue, and we believe that different subjects’ tongue will generally have different spectra due to differences health state. In order to see the differences between reflectance data more clearly, we firstly calculate the average of hyperviscosity patients healthy people, respectively. The average reflectance spectra of hyperviscosity subjects (as shown in blue dotted line) and healthy subjects (as shown in red solid line) are shown in Fig. 4. It is found that the average reflectance spectra in the 500–900 nm region for healthy subjects is higher than hyperviscosity subjects. Further, the normalize reflectivity scatterplots of each subject with the different wavelength are shown in Fig. 5. It is found that the convergence of the normalize reflectivity exist differences. Compared with other bands, there are greater difference between health and hyperviscosity in the 500–900 nm region. Therefore, we preferentially employ this region as the modeling data.

With Hold-out and Leave-one-out cross validations, the results of model are acquired, respectively. To measure the performance of the classification, sensitivity, specificity, accuracy and F-measure are calculated, respectively. In detail, *TP* is the number of true positive, *FP* is the number of false positive, *TN* is the number of true negative, *FN* is the number of false negative. Accuracy is expressed as (*TP *+ *TN*)/(*TP *+ *FP *+ *FN *+ *TN*). Sensitivity is true positive rate and is equal to *TP*/(*TP *+ *FN*), specificity is true negative rate and is equal to *TN*/(*TN *+ *FP*). F-measure is equal to *2TP*/(*2Tp *+ *FP *+ *FN*). And the results of two cross validations are as shown in Table 3.

**Table 3:** The evaluation of machine learning with Hold-out and Leave-one-out cross validation

Cross validation	Training set	Test set	Running times	Accuracy (%)	Specificity (%)	Sensitivity (%)	F-measure (%)
Hold-out	47	20	100	88.05	88.11	90.64	83.79
Leave-one-out	66	1	67	97.01	97.56	96.15	96.15

**Fig. 6:**
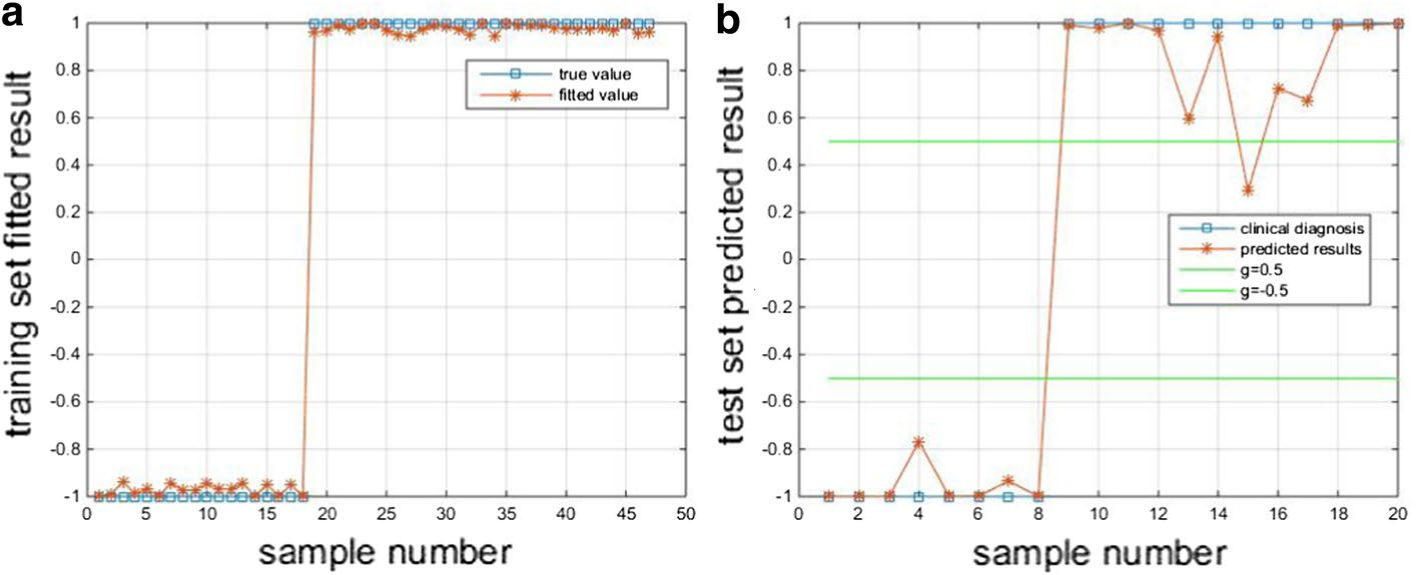
The optimized BP neural network classification model of Hold-out method. The asterisk represents the measured result and the square represents predicted result. **a** The training set outputs of the optimal model. **b** The test set outputs of the optimal model

The optimal model outputs of Hold-out cross validation method are as shown in Fig. 6. And the training set outputs of the optimal model is as shown in Fig. 6a, the test set outputs of the optimal model is as shown in Fig. 6b. Set deviation threshold (g) to 0.5, as shown in Fig. 6b, 95% of the absolute deviation are less than deviation threshold.

**Fig. 7:**
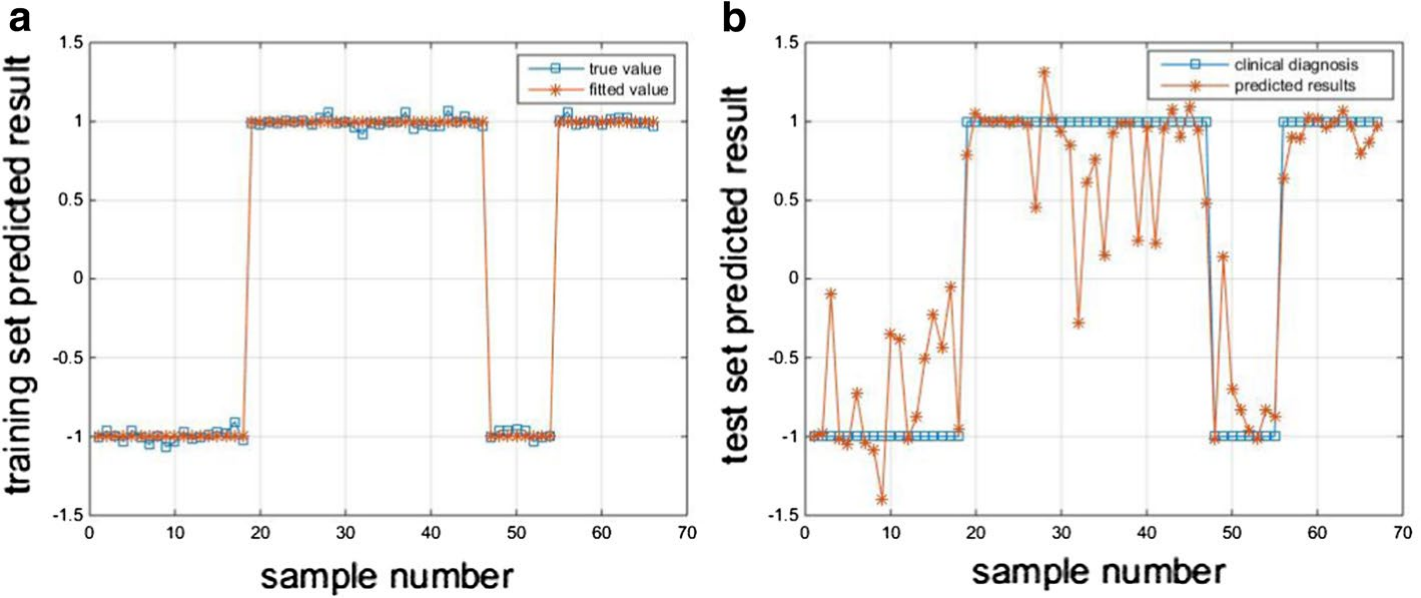
Test set classification based on the optimized BP neural network model. The square represents the measured result and the asterisk represents predicted result. **a** Hold-out the training set outputs of optimal model. **b** The 67 times outputs of the test set

The outputs of Leave-one-out cross validation method are as shown in Fig. 7. The training set outputs of optimal model is as shown in Fig. 7a, and the 67 times outputs of the test set is as shown in Fig. 7b. Set deviation threshold (g) to 0.5, as shown in Fig. 7b, 82.09% of the absolute deviation are less than deviation threshold.

**Fig. 8:**
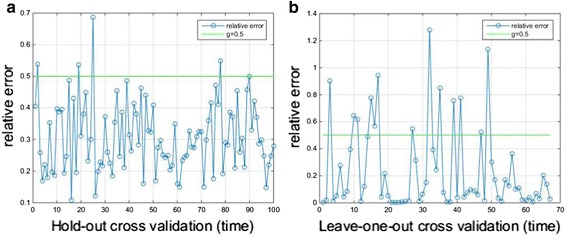
The relative error of Hold-out and Leave-one-out validations. **a** The relative error of Hold-out. **b** The relative error of Leave-one-out

Furthermore, the relative error of two cross validation methods is shown as Fig. 8. The relative error of Hold-out is as shown in Fig. 8a, and the relative error of Leave-one-out is as shown in Fig. 8b. Set the deviation threshold to 0.5, 96 and 80% of outputs are greater than the threshold with Hold-out method and Leave-one-out method, respectively.

## Discussions

In the process of subjects grouping, the clinical diagnosis results are provided by the experience physician primarily based on the hemorheology testing indices in China. Therefore, the testing indices comparing to other countries maybe slightly different.

In this study, we adopt PCA combined with ANN to verify whether it is feasible that hyperviscosity is identified with reflective spectroscopy of tongue tip. We choose PCA because there are mass data and redundancy in the spectrum. And PCA is a classic method of data extraction and compression in spectrum processing. Using PCA, we can find the most important and distinct basic spectrum to better build the classification model. We choose ANN because there is a certain nonlinearity between the tissue properties and reflective spectroscopy. And we also hope other machine learning methods are studies in this mission to find out the internal relation.

Cross validation is a way to avoid significant bias and lessen overfitting problem. In this study, two cross validations are used to model and assess the network modeling. 100 times validations are carried out with Hold-out method and 67 times validations are carried out with Leave-one-out method. From Table 3, it can be seen that the accuracy is 88.05 and 97.01%, respectively. And it indicates that the proposed method is able to classify the health and hyperviscosity with 67 subjects.

**Fig. 9:**
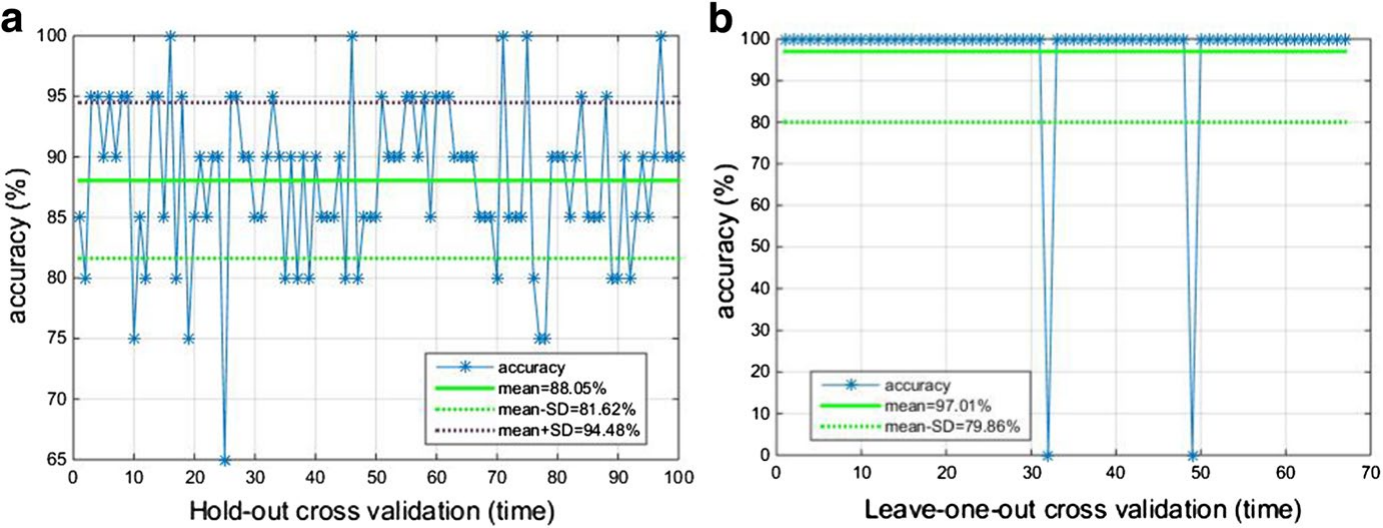
The accuracies of two cross validations. **a** The accuracies of 100 times Hold-out cross validation. **b** The accuracies of 67 times Leave-one-out cross validation. The blue asterisk represents the accuracy, the green line is the mean value of accuracies, the purple dash line is the mean value plus the standard deviation and the green dash line is the mean value minus the standard deviation

To estimate the stability of results, the accuracies of two cross validations are as shown in Fig. 9. Furthermore, the standard deviation (SD) and coefficient of variation (CoV) are calculated. SD and CoV of accuracies with 100 times Hold-out are 6.43 and 7.30%, respectively. For 67 times Leave-one-out, 2 prediction results are incorrect, and the rest results are correct. Therefore, SD and CoV of accuracies are 17.15 and 17.67%, respectively. It can be seen that the variability of accuracies with two cross validations are relatively stable, and the dispersion of the results are comparatively small.

## Conclusions

The purpose of this research is to develop a new noninvasive identification method of blood hyperviscosity disease. In this study, an experiment system is built and the reflectance spectra data is acquired at tongue tip from 67 male subjects. The spectra data is classified by combination with PCA and ANN data modeling, the analysis result of reflectance spectra data is contrast with blood sample analysis results. The experiment results show that tongue tip reflectance spectral analysis for healthy and blood hyperviscosity case classification can obtain good results with combination with PCA and ANN data modeling. The study indicates noninvasive determination of blood hyperviscosity is practicable with reflectance spectral analysis. In future experiments, the number of samples will be increased to enhance model robustness and classification ability.
